# ATM mutations in myelodysplastic neoplasms, acute myeloid leukemia, and myeloproliferative neoplasms: a systematic review

**DOI:** 10.1007/s12032-026-03378-4

**Published:** 2026-09-25

**Authors:** Lucas Oliveira Laurindo, Marjori Lima Boblitz Parente, Greyce Luri Sasahara, João Vitor Caetano Goes, Letícia Rodrigues Sampaio, Yuri Vasconcelos Pinheiro, Ronald Feitosa Pinheiro

**Affiliations:** 1 Cancer Cytogenomic Laboratory, Federal University of Ceara (UFC), Fortaleza, Ceara Brazil; 2 Drug Research and Development Center (NPDM), Federal University of Ceara, Fortaleza, Ceara Brazil; 3 Department of Clinical and Experimental Oncology (DOCE), Federal University of São Paulo, São Paulo, Brazil

**Keywords:** *ATM* mutations, Myeloid neoplasms, DNA damage, Genomic instability

## Abstract

Ataxia Telangiectasia Mutated (*ATM*), a central kinase in the DNA damage response, is recurrently altered across these disorders, including myelodysplastic neoplasms (MDS), acute myeloid leukemia (AML), and myeloproliferative neoplasms (MPN), yet its clinical significance remains underrecognized. We conducted a PROSPERO-registered systematic review (CRD420251142673) to evaluate the prognostic and biological impact of *ATM* alterations in MDS, AML, and MPN. Ten studies met inclusion criteria. In MDS, *ATM* alterations showed consistent downregulation, hypermethylation, and a predominance of oncogenic missense mutations, with methylation significantly enriched in cases progressing to AML. In AML, reduced *ATM* gene expression, often driven by miR-100 or miR-181a, was associated with increased blast proliferation, while the *ATM* genetic variant rs3092856 correlated with chemoresistance and inferior survival. Evidence in MPN/CML, although limited, suggests that *ATM* polymorphisms, such as rs228593, rs3092856 (C4138T), −5144A>T (rs228589), c.5753G>C, c.346A>G, c.4060C>A, and the germline variant L2307F, contribute to disease susceptibility and adverse prognostic stratification. Collectively, current evidence supports *ATM* alterations as clinically relevant biomarkers in myeloid malignancies, although their functional role in disease pathogenesis remains incompletely understood.. Rather than establishing *ATM* merely as a prognostic marker, future efforts should prioritize the characterization of *ATM*-related therapeutic vulnerabilities and integrate *ATM* status into treatment-oriented molecular panels to guide precision-based interventions in myeloid diseases.

## Introduction

The DNA is constantly affected by different types of damage from both endogenous and exogenous sources. The former includes reactive oxygen species [1], replication errors [2], hydrolysis and cellular aging [3, 4], while the latter comprises environmental factors such as chemical exposure [5, 6] and ultraviolet radiation [7]. Once DNA damage is detected, cells act like an orchestra composed of complex DNA repair mechanisms and checkpoint signaling pathways over the cell cycle phases to completely repair the lesion, whenever possible [8]. Among DNA repair systems in mammals, homologous recombination represents a main source of double strand breaks’ recovery. If not properly corrected, these lesions may induce genomic instability, resulting in chromosomal abnormalities and mutagenic events [9–11].

**Fig. 1:**
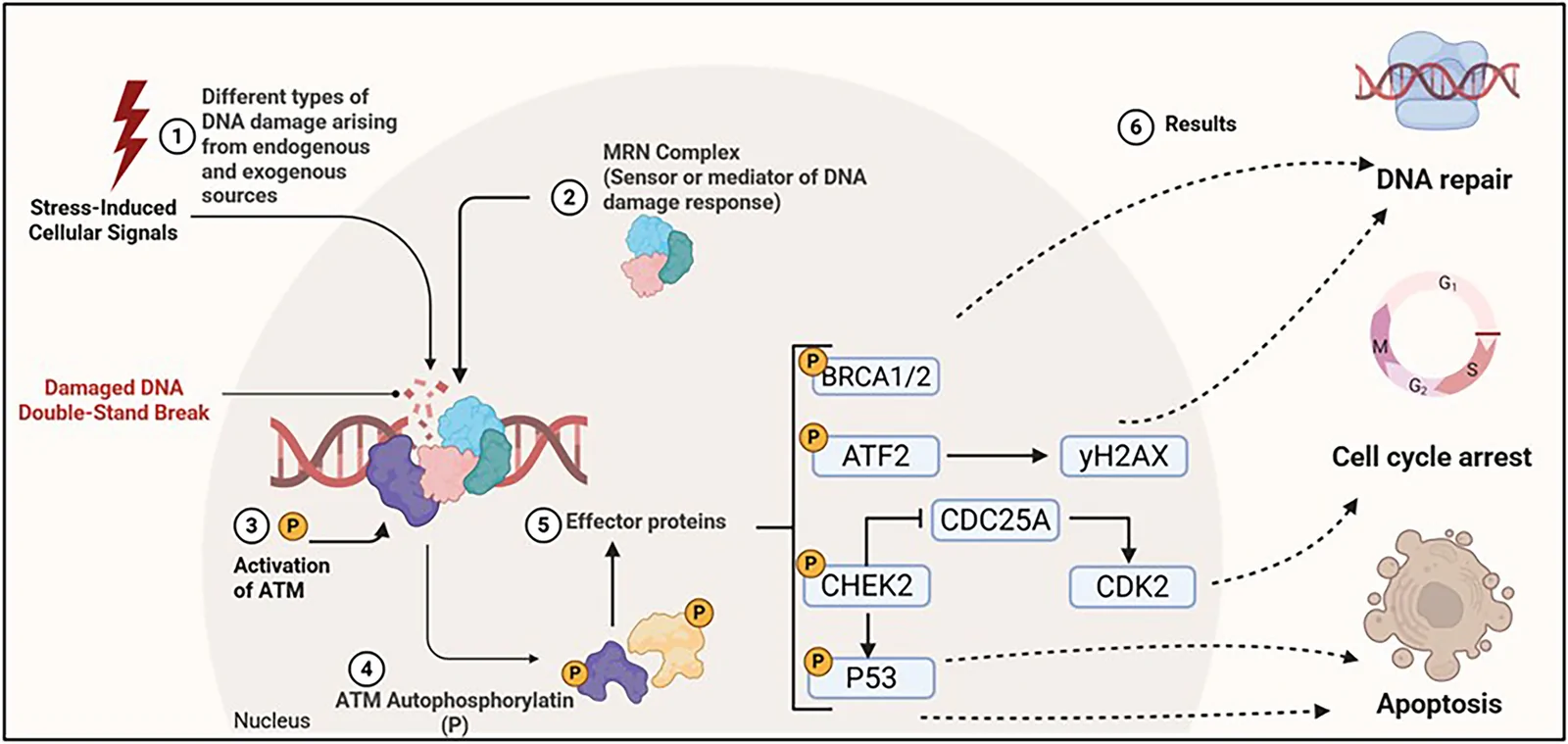
Activation mechanism of *ATM* kinase in response to DNA double-strand breaks (DSBs). Upon DDSBs induced by exogenous or endogenous stimuli, the MRN complex (MRE11–RAD50–NBS1) recognizes the insults and recruits ATM protein to the damage site. ATM protein then undergoes autophosphorylation, leading to its activation, and further phosphorylates multiple downstream targets. Among these, CHEK2 protein promotes inhibition of CDC25A, thereby suppressing CDK2 activity and inducing a transient cell cycle arrest, which in turn facilitates DNA repair. ATM protein also phosphorylates and stabilizes p53, both directly and through degradation of MDMX, enabling its tumor suppressor function. Activated p53 upregulates p21, which inhibits CDKs and enforces a cell cycle arrest at the G1/S or G2/M checkpoints, thereby preventing the transmission of unrepaired mutations. However, in cases of irreparable damage, p53 induces the transcription of pro-apoptotic genes such as *BID*, *BAX*, and *PUMA*, triggering programmed cell death. Image created using BioRender

In this scenario, the Ataxia-Telangiectasia Mutated (*ATM*) gene, located on chromosome 11q23.3 [12, 13], comprises 66 exons, encodes a serine/threonine protein active monomeric form of 370 kDa, and exerts a key role on DNA damage repair (DDR) and genomic stability [14]. In response to DNA lesions, especially in cases of DNA double-strand breaks (DSBs), a protein complex called MRN (composed by *MRE11, RAD50* and *NBS1* genes) identifies the insults, then recruits and activates the ATM protein at the damage site. Once activated, *ATM* signals and phosphorylate several downstream effectors such as *BRCA1, BRCA2* and *CHEK2*, among others [14, 15] (Fig. 1).

The role of *ATM* in cancer is multifaceted with germline and somatic mutations. Germline mutations in the *ATM* gene are classically associated with ataxia-telangiectasia (AT) syndrome, an autosomal recessive disorder characterized by genomic instability, immunodeficiency and increased predisposition to cancer. Still, several types of cancer have been associated with germline mutations in *ATM*, independent of AT syndrome, such as pancreatic, esophageal, lung, melanoma, breast, ovarian, prostate, and bladder malignant tumors [16], as well as in some lymphoma subtypes [17–19].

Regarding somatic mutations, *ATM* gene is usually a second hit after unrelated germline mutations in breast, ovary, pancreas, prostate and other tissues, but sometimes have been reported sporadically. More often, *ATM* somatic mutations are found amid hematological cancers, mostly on lymphoid malignancies, in which their frequency varies from 5–45% of cases. Among myeloid clonal disorders, incidence is much lower: less than 5% in acute myeloid leukemia (AML), 0.4% in myelodysplastic neoplasms (MDS) [20], the most common bone marrow cancer, and rarely between the chronic myeloproliferative subsets.

Overall, myeloid neoplasms represent a heterogeneous group of clonal hematologic disorders originating from genetic and epigenetic alterations in hematopoietic stem cells, that combined result in impaired hematopoiesis with a variable, but increased, risk of leukemic transformation, and protean clinical outcomes [21, 22]. Within the spectrum of these malignancies, MDS and AML are particularly prevalent, along with chronic myeloproliferative neoplasms (MPNs), which include chronic myeloid leukemia (CML), polycythemia vera (PV), essential thrombocythemia and primary myelofibrosis (PMF) [23].

*ATM* somatic and germline mutations, encompassing other gene deletions and/or epigenetic silencing, can disrupt DNA repair mechanisms, eventually promoting genomic instability that may lead to chromosomal aberrations—a common marker of clonal myeloid disorders—and disease pathogenesis. Moreover, *ATM* mutations can facilitate clonal evolution, inflict on adverse prognostic outcomes, leverage therapeutic resistance, and ultimately reduce survival. Therefore, investigating the clinical impact of *ATM* profile in myeloid disorders is of great relevance, not only for understanding the biological mechanisms underlying genomic instability, but also for identification of prognostic biomarkers and potential therapeutic targets. The characterization of these alterations may contribute to risk stratification in patients, guide personalized clinical decisions and open new perspectives in the field of precision medicine applied to hematology.

## Methodology

### Search strategy

This systematic review was submitted and registered in the International Prospective Register of Systematic Reviews (PROSPERO) database (CRD420251142673). We guided this study based on the PICO strategy (P-Population/Patient; I-Intervention; C-Comparison; O-Outcome/Result) [24] interested in answering the following question: “What is the relationship between genetic alterations in the *ATM* gene—including mutations, expression changes, and epigenetic modifications—and the prognosis in myeloid neoplasms (such as MDS, AML, CML, MPN)?”.

### Search database

We based this systematic review on the items in the Preferred Reporting Items for Systematic Reviews and Meta-Analyses (PRISMA 2020) to establish the minimum evidence required to conduct a systematic review of the role of the *ATM* gene in the evolution of pathogenesis, prognosis, and therapy among myeloid malignancies [25]. A search was conducted in the PubMed, SciELO, and LILACS databases to identify peer-reviewed, high-impact studies published between January 2010 and August 2025.

Studies written in Portuguese and English were included based on the following search terms and Boolean operators: "*ATM* gene" OR "ataxia telangiectasia mutated" AND "myelodysplastic syndrome" OR "MDS" OR "myeloproliferative neoplasms" OR "MPN" OR "polycythemia vera" OR "essential thrombocythemia" OR "primary myelofibrosis" OR "acute myeloid leukemia" OR "AML" OR "chronic myeloid leukemia" OR "CML".

The inclusion criteria for this study encompassed publications conducted in humans with hematological diseases that investigated the influence of the *ATM* gene on pathogenesis and outcomes. Only original research articles, including clinical, observational, cohort, and case–control studies were considered eligible.

Methodological quality and risk of bias were assessed qualitatively for each included study. The evaluation considered study design, sample size, major limitations and risk of bias. Studies were subsequently classified as low or moderate risk of bias according to their overall methodological robustness. Factors as small cohort size, lack of external validation, single-center recruitment, retrospective design, and heterogeneity in the assessment of *ATM* alterations, including gene expression, methylation, germline variants, somatic mutations, and polymorphisms were considered.

In addition, the search was restricted to articles published within the last 15 years. Reviews, meta-analyses, comments, perspectives, editorials, or other studies that did not provide original results or were unpublished were excluded. Two independent researchers reviewed the titles, abstracts, and full texts, with any conflicts resolved by a third researcher.

## Results

**Fig. 2:**
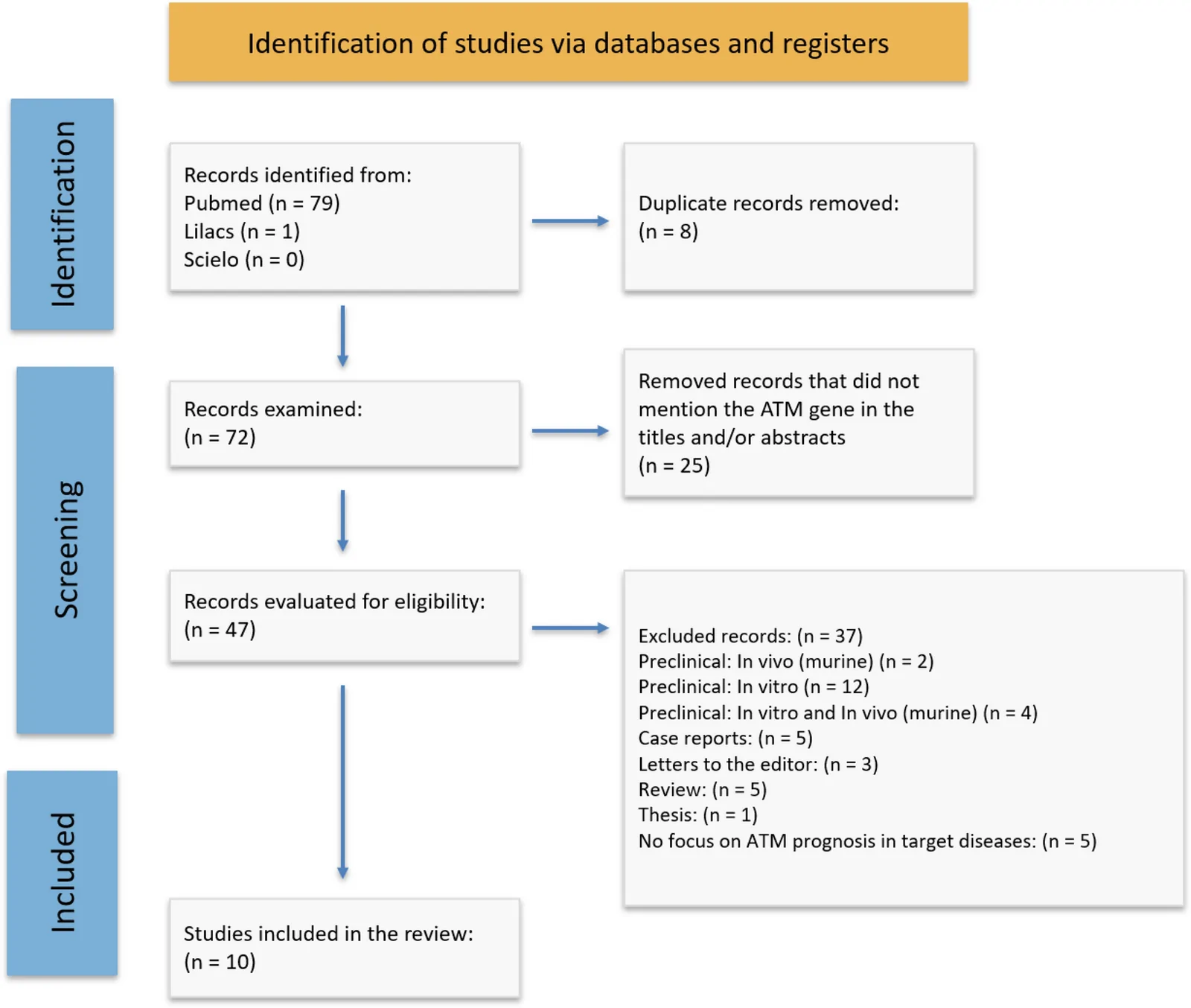
Flowchart of data obtained from the search of PUBMED/LILACS/SciELO records based on the PRISMA methodology

This systematic review identified 80 records (PubMed: 79; LILACS: 1; SciELO: 0). After removal of 8 duplicates (Fig. 2), 72 records remained for the first screening stage. Titles and abstracts were assessed to identify studies mentioning either the *ATM* gene or its description, resulting in the exclusion of 25 articles. During the second screening stage, following the PRISMA guidelines, 37 additional records were excluded: 2 preclinical murine studies, 12 preclinical in vitro studies, 4 preclinical studies conducted both in vitro and in murine models, 5 case reports, 3 letters to the editor, 5 reviews, 1 thesis, and 5 studies addressing other diseases. Ultimately, 10 articles met the eligibility criteria and were included in this systematic review, with the study design, disease, sample size, population, outcome measures, mais findings and risk of bias summarized in Table 1.

**Table 1:** Summary of the selected studies investigating the role of *ATM* in myeloid malignancies

Study design	Disease	Sample size	Population	Outcome measures	Main findings	Risk of bias	Ref
Analytical cross-sectional	MDS	232 patients (Cohorts A: 56, B: 100, C: 76) and healthy controls	Adult patients with Myelodysplastic Neoplasm (MDS)	Methylation/mutation status of DNA repair genes, *ATM* expression, and evolution to AML	Higher *ATM* methylation in MDS patients progressing to AML; reduced *ATM* expression in MDS regardless of IPSS-R risk; predominance of missense, likely oncogenic *ATM* mutations; *ATM* frequently silenced/downregulated	Moderate	Pinheiro et al. [20]
Prospective cohort	MDS	31 patients	Younger adult patients (aged 18–60 years) with de novo MDS	Frequency of germline predisposing variants via Exome Sequencing and Targeted NGS	Germline heterozygous *ATM* variants (all VUS) detected in low-risk MDS; no associations with survival, transfusion needs, or marrow features	Moderate	Attardi et al. [27]
Analytical cross-sectional	MDS	47 patients	Adult patients with MDS	mRNA expression levels of HR and NHEJ genes (*ATM, XRCC6, LIG4*) and associations with polymorphisms	Functional polymorphisms (rs228593, rs2267437, rs1805388) significantly alter the expression levels of *ATM, XRCC6*, and *LIG4*, driving genomic instability in MDS	Moderate	Ribeiro et al. [28]
Analytical cross-sectional	MDS	51 patients	Adult de novo MDS patients	Expression of NER, HR, and NHEJ genes (*ATM, BRCA1, LIG4, XPA*) related to bone marrow cellularity and survival	Hypocellular MDS patients have decreased expression of *ATM, BRCA1/2, LIG4*, and *ERCC8*	Moderate	Ribeiro et al. [29]
Retrospective cohort	MDS	109 cases (74 MDS, 15 AML, 20 controls)	Adult de novo MDS, de novo AML patients, and reactive bone marrow controls	Expression of DDR proteins (pNBS1, pATM, γH2AX, p53) and overall survival (OS)	DDR proteins (pNBS1, pATM, γH2AX) were significantly increased in MDS and AML	Moderate	Kefala et al. [30]
Analytical cross-sectional with in vitro analysis	AML	53 clinical subjects (30 AML, 23 controls) and cell lines	Pediatric AML patients and myeloid leukemia cell lines (Kasumi-1 and MV-4-11)	miR-100/*ATM* expression, cell viability (MTT assay), and apoptosis	mRNA level of *ATM* was lower in AML group than that of normal group. Moreover, a notable downregulation of *ATM* was also observed in AML cells	Moderate	Sun et al. [31]
Analytical cross-sectional with in vitro analysis	AML	75 subjects (57 pediatric AML, 18 controls) and cell lines	Pediatric AML patients and myeloid leukemia cell lines (NB4, HL60, MV-4-11, THP1, K562, CCRF-CEM, Jurkat, and SUP-B15)	miR-181a/*ATM* expression, cell proliferation, and cell cycle (G1/S transition)	*ATM* expression was reduced in pediatric AML and inversely correlated with miR-181a levels. *ATM* was as a direct target of miR-181a, and its suppression promoted cell proliferation and G1/S cell cycle progression	Moderate	Liu et al. [32]
Case–control	AML	867 subjects (307 AML, 560 controls)	Chinese patients with newly diagnosed AML and healthy controls	42 genetic variations in 15 DNA repair genes; chemotherapy response and OS	*ATM* 4138C>T (rs3092856) variant linked to higher chemotherapy refractoriness; associated with reduced overall survival (median 7 vs. 11 months); CT genotype showed OS <2 years, while CC genotype had better survival	Low	Shi et al. [33]
Case–control	CML	925 subjects (476 CML, 449 controls)	Chronic Myeloid Leukemia (CML) patients from India	*ATM* gene polymorphisms (− 5144A>T and C4138T), EUTOS score, and event-free survival	The A allele of − 5144A>T (rs228589) and T allele of C4138T (rs3092856) are significantly associated with enhanced risk for CML development and progression, correlating with high-risk prognostic EUTOS scores	Low	Gorre et al. [34]
Case–control with in vitro analysis	MPN	848 patients (64 familial, 218 unselected, from a registry of 630) and cell lines	Patients with Myeloproliferative Neoplasms (MPNs) and myeloid cell line TF1	Recurrent germline variants, structural *ATM* modeling, and downstream CHEK2 phosphorylation	Identified a recurrent *ATM* L2307F germline variant in ~8% of familial MPNs. The variant stabilizes inactive *ATM* and decreases *CHEK2* phosphorylation, increasing MPN predisposition	Moderate	Braunstein et al. [35]

The Table 1 included studies demonstrated low to moderate risk of bias. Two studies were classified as having low risk of bias due to their large sample sizes and robust observational designs, whereas the remaining studies were considered to have moderate risk of bias, mainly because of limited cohort sizes, single-center recruitment, lack of validation cohorts, and methodological heterogeneity.

Most investigations evaluated *ATM* alterations through distinct methodologies, including gene expression analyses, DNA methylation profiling, polymorphism screening, germline variant identification, and next-generation sequencing approaches. This diversity limited direct comparisons across studies and precluded quantitative synthesis of the available data. Overall, the current evidence should be interpreted as low-to-moderate quality and primarily hypothesis-generating.

### Myelodysplastic neoplasms (MDS)

Five articles qualified for the MDS subset, the most frequently reported clonal myeloid disorder associated with *ATM*:

Pinheiro et al*.* [20] evaluated *ATM role in MDS* regarding its methylation status, gene expression and mutational profile in 232 patients with MDS distributed into three different cohorts, using a wide methodology, including pyrosequencing, real-time PCR, immunohistochemistry, and NGS-Sanger. Among all genes involved with DSBs repair, *ATM* displayed higher methylation patterns among patients that evolved to acute myeloid leukemia: those who progressed exhibited twice the methylation rate when compared to those who did not progress to AML (median 4.97 versus 2.45; p = 0.016). Likewise, MDS patients presented downregulation of *ATM*, including significant reductions of *ATM* expression when compared to elderly (non-MDS) controls, regardless of the disease risk classification (according to the IPSS-R score).

Regarding the mutational profile of *ATM*, the same authors [20] investigated 7583 MDS cases from cBioportal, composed of three datasets: Myelodysplasia (UTokyu, Nature 2011, n = 29), Myelodysplastic (MSK, 2020, n = 4231), and the most recent 3323 cases from the Molecular International Prognostic Score System (Molecular IPSS). A great number of mutations was detected on *ATM*, with frameshifts and missenses standing out above other types, while being mostly categorized as likely oncogenic/oncogenic according to the gold-standards for classification of somatic variations in cancer [26]. The authors eventually concluded that *ATM* is frequently silenced or downregulated in MDS either due to methylation or mutations, what underscores its potential role as a future target for therapeutic intentions.

In another recent study, Attardi et al*.* [27] investigated an unusual cohort of 31 young (< 60 years old) low-risk MDS patients presenting with neutropenia and thrombocytopenia, and also a recent disease onset, using next generation sequencing (NGS) on samples obtained from peripheral blood and saliva. The authors explored the profile of 344 genes, including *ATM*, looking for germline variants linked to clonal and non-clonal cytopenia and encountered only heterozygous variants in *ATM* (c.5753G>C, c.346A>G, c.4060C>A), all classified as variants of undetermined significance (VUS). No significant associations were detected in respect of survival, mortality, transfusion requirements, or bone marrow morphological features.

Ribeiro et al*.* evaluated *ATM* polymorphisms and expression on 47 and 51 MDS patients, respectively [28, 29] Authors first reported [28] that SNP rs228593 (genotypes AA or AG + AA) was associated with increased *ATM* expression, particularly among patients classified as high- or very-high-risk leukemic progression by the IPSS-R. Among genotypes, AA patients displayed the highest expression levels when compared to GG cases (wild-type). In the next study [29], authors found that *ATM* expression was significantly reduced in the group of patients with hypocellular MDS, yet no relevant correlations could be observed in terms of survival, mortality, transfusion needs, or other hematological parameters. Both findings indicate that: 1) rs228593 is a functional *ATM* polymorphism in MDS, and 2) AA genotype may be linked to adverse prognosis.

Similarly, Kefala et al*.* [30] evaluated the expression of DDR proteins, including *ATM* (pNBS1, γH2AX, and pATM) by immunofluorescence and immunohistochemistry in 74 patients with MDS, but no significant differences showed up in terms of survival or hematological parameters regarding pATM.

### Acute myeloid leukemia (AML)

Three articles qualified for AML analysis, two on pediatric and one on adult cohorts:

Sun et al*.* [31] studied the expression of miR-100 and *ATM* by qRT-PCR in 30 pediatric AML patients (0–14 years). The authors reported that reduction of *ATM* expression was associated with increased G1/S transition on the cell cycle, resulting in greater proliferation of myeloblasts.

Liu et al. [32] also evaluated the expression of miR-181a and *ATM* by qRT-PCR in 57 pediatric AML cases (mean age 7 years) and detected a very similar underexpression of *ATM* among patients.

Shi et al. [33] studied 307 AML patients (mean age 42 years), including primary and secondary AML. DNA from bone marrow (patients) and peripheral blood (controls) was genotyped using MALDI-TOF MS for 42 SNPs in 15 DNA repair and damage response genes. The *ATM* 4138C>T (rs3092856) variant was associated with higher chemotherapy refractoriness (7/8 cases) and lower overall survival (median 7 vs. 11 months). The CT genotype was associated with reduced overall survival of less than two years, while for the CC genotype 38% showed superior outcome. So far, this is the largest cohort studied about the influence of *ATM* polymorphisms in AML.

### Myeloproliferative neoplasms (CML, PV, essential thrombocythemia and primary myelofibrosis)

Research on the *ATM* in the context of CML and MPN is scarce. Our review identified two studies:

Gorre et al. [34] analyzed 476 CML patients (mean age 51) for *ATM* polymorphisms (− 5144A>T, rs228589, and C4138T, rs3092856), assessing genotype/allele frequencies, haplotypes and risk models; and correlating with traditional prognostic scores (like Sokal, Hasford and EUTOS), survival and response to imatinib. *ATM* variants were associated with higher CML risk and correlated EUTOS scores.

In the remaining study, Braunstein et al. [35] evaluated familial myeloproliferative neoplasms (ET, PV, PMF) in 64 individuals (mean age 49.7) using whole-genome sequencing of peripheral blood to identify recurrent germline predisposition variants. The authors detected a VUS in *ATM* (L2307F) among some cases, but with no genomic instability amid the disease subtypes.

## Discussion

This review depicts that research is still limited in the field of *ATM* pathogenesis among clonal myeloid disorders. Encoding a PI3/PI4-kinase family protein that regulates multiple downstream steps involved in cell regulation/fate, this analysis of only 10 eligible studies could still unveil divergent findings within the different myeloid neoplasms, reflecting both the complexity of *ATM* biology and, of utmost importance, the very limited number of clinical studies addressing its role and/or prognostic impact in these malignancies.

Most studies in MDS depict reduced *ATM* expression or hypermethylation, pointing towards a downregulated *ATM* pathway in its pathogenesis. However, the prognostic significance of these alterations remains controversial, since several studies failed to demonstrate clear correlations with survival, association to classic cytogenetic risk factors, or interplay within the DDR machinery.

In AML, particularly on pediatric cohorts, studies indicated that *ATM* downregulation promoted cell proliferation and impaired differentiation, often regulated through miRNAs such as miR-181a and miR-100 [36]. MicroRNAs (miRNAs) are small non-coding RNAs that regulate gene expression post-transcriptionally and play critical roles in hematopoiesis and leukemogenesis. In AML, dysregulated miRNA expression profiles have been associated with disease classification, therapeutic response, and prognosis.

According to Bräuer-Hartmann et al. [37, 38], the miR-181a/b microRNA cluster, which is triggered by the PML/RARα fusion, suppresses the tumor suppressor RASSF1A, thereby preventing granulocytic differentiation and promoting cell proliferation in acute promyelocytic leukemia (an AML unique subtype). Treatment with all-trans-retinoic acid lowers miR-181a/b levels, restores RASSF1A expression, and leads to cell cycle arrest, apoptosis, and differentiation. These findings highlight the importance of miRNAs as post-transcriptional regulators of tumor suppressor genes and as potential therapeutic targets in myeloid, suggesting that *ATM* is subject to multiple layers of regulation, each with distinct implications for AML pathogenesis.

Data on CML and MPN are very limited. One study found that *ATM* polymorphisms (− 5144A>T, rs228589 and C4138T, rs3092856) were linked to higher CML risk and worse prognostic scores, while another found an *ATM* variant of uncertain significance (L2307F). The SNP 4138C>T, previously linked to CML prognosis, was also found to be significantly associated with chemotherapy resistance and reduced survival in AML [33].

While studies on *ATM* remain scarce in the hematology field, intragenic methylation of *ATM* has been identified in peripheral white blood cells from breast cancer patients, supporting the hypothesis that epigenetic alterations may precede genetic mutations during malignant transformation [39]. In the same way, hypermethylation of *ATM* has been linked to oncogenesis and prognosis in solid tumors, such as breast cancer [40].

This review has limitations, including the restricted number of eligible articles, heterogeneity among studies regarding design, sample size and laboratory methods, as well as the lack of functional analyses for many *ATM* mutations or polymorphisms. Publication biases, reliance on retrospective studies, and population variability also limit inferences and projections. Thus, *ATM* warrants further investigation in myeloid malignancies, to demystify its putative role in regulating chromosomal abnormalities, a hallmark of conditions like MDS, AML, CML, and PV.

The heterogeneity of findings likely reflects methodological differences among studies. MDS studies evaluated distinct *ATM*-related alterations, including methylation, gene expression, protein expression, germline variants, and somatic mutations. Furthermore, sample sizes ranged from 31 to 232 patients, and outcomes varied from leukemic transformation to survival and transfusion dependence. Similarly, AML studies included both pediatric and adult populations and assessed either *ATM* expression or genetic polymorphisms. These differences limit direct comparisons and may explain the inconsistent prognostic associations observed across studies.

Future research should prioritize multicenter studies with standardized methods, include *ATM* status in molecular panels, and conduct functional analyses to clarify its role in leukemogenesis. Furthermore, assessing *ATM* from a therapeutic perspective might also be relevant, especially with novel DNA repair inhibitors and synthetic lethality approaches.

## Data Availability

No datasets were generated or analysed during the current study.
